# Implications of a *De Novo* Variant in the *SOX12* Gene in a Patient with Generalized Epilepsy, Intellectual Disability, and Childhood Emotional Behavioral Disorders

**DOI:** 10.3390/cimb46070383

**Published:** 2024-06-26

**Authors:** Simone Treccarichi, Francesco Calì, Mirella Vinci, Alda Ragalmuto, Antonino Musumeci, Concetta Federico, Carola Costanza, Maria Bottitta, Donatella Greco, Salvatore Saccone, Maurizio Elia

**Affiliations:** 1 Oasi Research Institute-IRCCS, 94018 Troina, Italy; streccarichi@oasi.en.it (S.T.); fcali@oasi.en.it (F.C.); mvinci@oasi.en.it (M.V.); aragalmuto@oasi.en.it (A.R.); amusumeci@oasi.en.it (A.M.); mbottitta@oasi.en.it (M.B.); dgreco@oasi.en.it (D.G.); melia@oasi.en.it (M.E.); 2 Department of Biological, Geological and Environmental Sciences, University of Catania, Via Androne 81, 95124 Catania, Italy; concetta.federico@unict.it; 3 Department of Sciences for Health Promotion and Mother and Child Care “G. D’Alessandro”, University of Palermo, 90128 Palermo, Italy; carola.costanza@unipa.it

**Keywords:** next generation sequencing, *SOX12* gene, epilepsy, neurodevelopmental delay

## Abstract

*SRY-box transcription factor* (*SOX*) genes, a recently discovered gene family, play crucial roles in the regulation of neuronal stem cell proliferation and glial differentiation during nervous system development and neurogenesis. Whole exome sequencing (WES) in patients presenting with generalized epilepsy, intellectual disability, and childhood emotional behavioral disorder, uncovered a *de novo* variation within *SOX12* gene. Notably, this gene has never been associated with neurodevelopmental disorders. No variants in known genes linked with the patient’s symptoms have been detected by the WES Trio analysis. To date, any MIM phenotype number associated with intellectual developmental disorder has not been assigned for *SOX12*. In contrast, both *SOX4* and *SOX11* genes within the same C group (*SoxC*) of the *Sox* gene family have been associated with neurodevelopmental disorders. The variant identified in the patient here described was situated within the critical high-mobility group (HMG) functional site of the *SOX12* protein. This domain, in the Sox protein family, is essential for DNA binding and bending, as well as being responsible for transcriptional activation or repression during the early stages of gene expression. Sequence alignment within *SoxC* (*SOX12*, *SOX4* and *SOX11*) revealed a high conservation rate of the HMG region. The in silico predictive analysis described this novel variant as likely pathogenic. Furthermore, the mutated protein structure predictions unveiled notable changes with potential deleterious effects on the protein structure. The aim of this study is to establish a correlation between the *SOX12* gene and the symptoms diagnosed in the patient.

## 1. Introduction

The history of the *SRY-box transcription factor* (*SOX)* gene family dates back to its discovery in 1990 through the cloning of the mammalian *sex-determining region Y* (*Sry*) gene, codifying the testis-determining factor (TDF). This breakthrough was achieved by identifying conserved DNA sequences on the Y chromosomes of other mammals [1,2,3]. At the onset of the 21st century, a comprehensive enumeration and classification of *SOX* genes was undertaken. Through meticulous analysis of the initial whole genome sequences available, researchers identified a total of 20 *SOX* genes in both mice and humans [4]. *SOX* genes are involved in a wide range of developmental processes, including the development of the nervous system, the skeleton, the cardiovascular system and the male and female reproductive systems. Furthermore, they govern the maintenance of the pluripotency of stem cells, cell proliferation and cell fate decisions/germ layer formation as well as terminal cell differentiation into tissues and organs [5,6].

As documented, *SOX* transcription factors (TFs) act as important regulators of neuronal stem cell proliferation and glial differentiation during nervous system development and adult neurogenesis [6]. They contribute to the formation of neural circuits, thus playing a key role in establishing neuronal connectivity within the nervous system [7,8]. *SOX* TFs are crucial for neural progenitor identity, expressed from its onset and persisting throughout development and adulthood. Research into mammals’ systems underscores their role in promoting self-renewal and maintaining progenitor cell potency, enabling both proliferation and differentiation [9]. In particular, Sox transcription factors like *SOX2*, *SOX21*, *SOX4* and *SOX11* regulate crucial aspects of adult neurogenesis such as radial glial cell maintenance, intermediate progenitor cell proliferation, newborn neuron maturation, axonal growth and dendritic morphogenesis with subsequent neuronal migration within the developing cerebral cortex [10,11,12,13,14].

*SOX* proteins exhibit both the characteristics of a classical transcription factors and chromatin architectural components. In fact, they interact with other transcription factors, facilitating DNA binding and bending, thereby modulating transcriptional activation or repression during the early stages of gene expression [15,16]. Dysregulation of *SOX* genes can lead to neurological disorders and diseases, highlighting their importance in neural development and function. In fact, *SOX* proteins act as pioneering regulators, occupying silenced target genes and keeping them in a poised state for activation during subsequent phases of differentiation [6,17]. As has been extensively documented, mutations in *Sox* genes, specifically *SOX1*, *SOX2* and *SOX11*, are implicated in the onset of epileptic seizures in both human patients and animal models [18,19,20,21,22]. It has been demonstrated that diverse *SOX* genes are crucial during cancer development. In fact, their plausible use in cancer therapeutics has been postulated [23,24].

The *SOX* gene family comprises several groups, each with distinct functions. For instance, the B1 group, represented by *SOX1*, *SOX2* and *SOX3*, plays critical roles in early embryonic development and neural stem cell maintenance. Additionally, the B2 group, represented by *SOX14* and *SOX21*, is implicated in various developmental processes, including eye and brain development. The C group of *SOX* genes, consisting of *SOX4*, *SOX11* and *SOX12*, is pivotal in various developmental processes, particularly neurogenesis. These genes play essential roles in regulating cell fate determination and differentiation within the nervous system [25,26]. The D group, including *SOX5*, *SOX6* and *SOX13*, is involved in neural and skeletal development. Moreover, according to entries in the OMIM database, 11 *SOX* genes have been assigned unique entry codes.

Within the C group, *SOX12* stands out as the only member that has not been assigned a MIM phenotype number associated with intellectual developmental disorder. Although it does have an OMIM entry (601947), it is not linked to any disease. As already documented, *SOX12* is crucial for visual pathway development [27]. Furthermore, *SOX12* has been identified as potential target for acute myeloid leukemia [28]. Knockdown of *SOX12* expression inhibits the proliferation and metastasis of lung cancer cells [29]. Moreover, it has been found to be overexpressed in colorectal cancer [30].

The *SOX*-C genes exhibit redundant expression patterns [25], with *SOX4* and *SOX11* displaying extensive overlap in embryonic expression. In contrast, *SOX12* shows more uniform expression levels without specific high-expression sites. Moreover, all three proteins demonstrate similar DNA-binding characteristics and function as transcriptional activators [31]. As is known, *SOX4*, *SOX11* and *SOX12* are all expressed in the post-mitotic neurons of the central nervous system [25,32]. Specifically, all of them participate in the regulation of neuronal maturation during embryonic neurogenesis [32,33,34]. As outlined, *SOX12* has been identified as a candidate gene involved in developmental delay in a microdeletion of 20p13, which includes *SOX12* along with another gene [35]. *SOX12* plays a critical role in organogenesis, facilitating the transition of pluripotent embryonic stem cells into multipotent neural and mesenchymal cells. These progenitor cells are essential for embryo growth and organ development, as they possess the capacity for self-renewal and differentiation into various cell types. As reported, *SOX12* has not been detected in the adult mouse brain [36].

The absence of detailed studies on *SOX12* in relation to epilepsy, intellectual disability and childhood emotional behavioral disorders represents a critical gap in our knowledge. Understanding whether variants in *SOX12* contribute to these conditions could provide new insights into their genetic underpinnings and potentially lead to novel therapeutic strategies. In this study, we aim to establish a potential link between a *de novo* variant identified within the *SOX12* gene through WES and epilepsy, diagnosed in the patient under examination. Furthermore, our investigation seeks to address this gap in the literature by exploring the potential involvement of *SOX12* in neurodevelopmental processes, thus laying the groundwork for future research into the diverse array of genes implicated in epilepsy and neurodevelopmental disorders.

## 2. Materials and Methods

### 2.1. Library Preparation and NGS Analysis

Genomic DNA was extracted from peripheral blood leukocytes obtained from the patient and both parents. DNA extraction was conducted as previously described [37]. Library preparation (TRIOS) and exome enrichment was carried out using the Agilent SureSelect V7 kit (Santa Clara, CA, USA) according to the manufacturer’s instructions. A sequencing run was performed on an Illumina HiSeq 3000 instrument (San Diego, CA, USA). This approach achieved 97% of regions covered at a minimum of 20× magnification. We filtered the identified variants according to (i) recessive/*de novo*/X-linked pattern of inheritance and (ii) allele frequencies (mean average frequency, MAF) <1%, using as reference the following genomic datasets: 1000 Genomes, ESP6500, ExAC, gnomAD. Integrated Genomics Viewer (IGV) [38] was used to display DNA sequences. To confirm the identified mutation, Sanger sequencing was performed using the BigDye Terminator v1.1 Cycle Sequencing Kit (Life Technologies, Carlsbad, CA, USA) with an ABI 3130 instrument (Life Technologies, CA, USA) as previously described [39]. Primers were for. 5′-CGGCGGAAGATCATGGACCAGTG-3′, rev. 5′-GCGGCCCGGGCTTGAG-3′.

### 2.2. Data Analysis

The Uniprot database (https://www.uniprot.org/) (accessed on 15 March 2024) was used for retrieving the SOX12 protein details related to the functional regions and domains. Additionally, the Uniprot alignment tool was used for obtaining the percentage of identity of *SOX12* with each SOX protein. *SOX12* expression among the different tissue was retrieved on the specific expression databases Genotype-Tissue Expression (GTEx) (https://www.gtexportal.org/) [40] (accessed on 15 March 2024) and the Human Protein Atlas (https://www.proteinatlas.org/) [41] (accessed on 15 March 2024). The amino acid structure of SOX12 protein was retrieved from the UCSC Genome Browser (https://genome.ucsc.edu/) (accessed on 15 March 2024) database. Gene ontology terms (GO) related to the functional high-mobility group (HMG) domain annotation were retrieved from the QuickGO database (https://www.ebi.ac.uk/QuickGO/) [42] (accessed on 15 March 2024). BioEdit software version 7.2 was used for retrieving the graphical representation of the sequence alignment [43]. Protein structure predictions were generated through AlphaFold prediction algorithms based on the machine learning program DeepMind Technologies (London, UK), employing UCSF ChimeraX software version 1.7 (software developed by the Resource for Biocomputing, Visualization and Informatics at the University of California, San Francisco, with support from the National Institutes of Health R01-GM129325 and the Office of Cyber Infrastructure and Computational Biology, National Institute of Allergy and Infectious Diseases) (https://www.cgl.ucsf.edu/chimerax/), (accessed on 15 March 2024) as described previously [44]. Notably, the AlphaFold algorithm generated five models, and in accordance with its output, the “best model” was selected for this investigation. In the Supplementary Materials, the procedure for the selection of the best model is clearly depicted and precisely described in Figures S2 and S3.

The pathogenic variants were investigated in the Human Gene Mutation Database (HGMD Professional 2023). Diverse VarAFT [45] filters were employed on the vcf files. The observed variant was described according to the American College of Medical Genetics (ACMG) guidelines [46] indicated in Table 1 and was actualized with VarSome according to a previous study [47] and other evidence from the literature. PhastCons100way and PhyloP100way scores (from VarSome analysis) were used for analyzing the conservation tendency of the specific mutation region.

The in silico analysis employing multiple algorithms was performed via the VarSome platform release 11.12, employing diverse tools including Mutation Taster 2021 (https://www.mutationtaster.org/) (accessed on 15 March 2024) and MutPred v.2.0 (http://mutpred2.mutdb.org/) (accessed on 15 March 2024) [48], as well as FATHMM (https://fathmm.biocompute.org.uk/) (accessed on 15 March 2024), and FATHMM-MKL v.2.3 (https://fathmm.biocompute.org.uk/) (accessed on 15 March 2024). According to a previous work, FATHMM scores around zero suggest no significant change in amino acid probabilities, while scores below zero indicate an unfavorable substitution, with the mutant residue less likely than in the wild type. Scores above zero suggest a favorable substitution, with the mutant residue more likely than the wild type [49]. Conversely, FATHMM-MKL score is a predictive value used to assess the functional impact of genetic variants on protein function, particularly focusing on the likelihood of pathogenicity. Its value ranges from 0 to 1 [50]. The MuPRO v.12.0 (http://mupro.proteomics.ics.uci.edu/) (accessed on 15 March 2024) tool was used to predict the value and sign of energy change (delta delta G) caused by mutations, with a confidence score ranging from −1 to 1. Scores below 0 indicate decreased protein stability, with lower scores indicating higher confidence, while scores above 0 indicate increased stability, with higher scores signifying greater confidence [51]. For the MuPRO analysis, the input inserted was the amino acid sequence of the protein, specifying the mutation site. The analysis of the proteins involved in the same *SOX12* pathways was carried out employing the databases STRING 12.0 (https://string-db.org/) [52] (accessed on 15 March 2024), BioGRID v.4.4.234 (https://thebiogrid.org/) [53] (accessed on 15 March 2024) and IntAct portal v.10.4 (https://www.ebi.ac.uk/intact/) [54] (accessed on 15 March 2024).

The percentages of protein sequence identity for the 19 Sox genes were obtained from the Uniprot database using the Align tool release 2024_03. Heatmaps and dendrograms were generated in RStudio version 3.6.3 using the ggplot2, gplots, ggdendro and tidyverse packages.

## 3. Results

### 3.1. Clinical Report

The patient was a 16-year-old male. There was no known family history of febrile seizures, epilepsy or other neurological conditions. Both parents were healthy and non-consanguineous. Pregnancy was uneventful. Born at term, at birth his weight was 3850 g; length and cranial circumference were unknown. There were no reported jaundice or asphyxia. The patient’s developmental milestones were reported as normal. He achieved appropriate developmental milestones for his age, including sitting, crawling, walking and language acquisition, without any significant delays or regressions.

At the age of 6, he started to present absence seizures. These episodes were characterized by sudden staring spells lasting approximately 10 to 20 s. The seizures occurred many times a day (2–3/day), significantly impacting the patient’s daily activities and school performance.

He was brought to our observation at 7 years of age. Phenotype was characterized by coarse facial appearance, widow’s peak hairline, broad and anteverted nasal tip, thick lips, microretrognathia, low tongue posture, single palmar crease on the right hand, diffuse muscle hypotonia and hyperlaxity of ligaments. Interictal EEG showed generalized spike- and polyspike-and-wave complexes, prevalent over the frontotemporal regions of both hemispheres. Ictal EEG during wakefulness and drowsiness was characterized by generalized discharges of spike-and-wave complexes at 3.5–4 Hz of variable duration (2–14 s). Brain MRI did not reveal any malformations or focal parenchymal lesions but showed a slight volumetric predominance of the left lateral ventricle. Cortical sulci were moderately and symmetrically accentuated. CGH array analysis, performed at the age of 13 years, did not detect any genomic imbalances with a probable pathogenic role. Three years later, whole exome sequencing analysis was performed. This was carried out in the frame of the second hospitalization of the patient.

The patient was initially treated with valproic acid. However, the effectiveness of the medication in controlling absence seizures was limited, and levetiracetam was added. Since the patient showed irritability and poor tolerance to frustration, levetiracetam was replaced by ethosuximide. At present, he is taking ethosuximide (600 mg/day) and sodium valproate (900 mg/day), and absences have been completely controlled since the age of 9. Interictal EEG shows sporadic brief bursts of spike-and-wave complexes over the parietal-temporal–occipital regions of both hemispheres and sporadic sharp waves over the parietal regions.

At 10 years of age, behavioral disturbances emerged, characterized by oppositional conduct, poor adherence to rules and fixed thoughts. Sporadic episodes of anxiety, with somatization, vomiting, diurnal enuresis and poor socialization with peers were described. At the last observation, at 16 years of age, a diagnosis of mild intellectual disability and emotional behavioral disorder in childhood and adolescence was made.

### 3.2. Genetic Analysis

**Figure 1:**
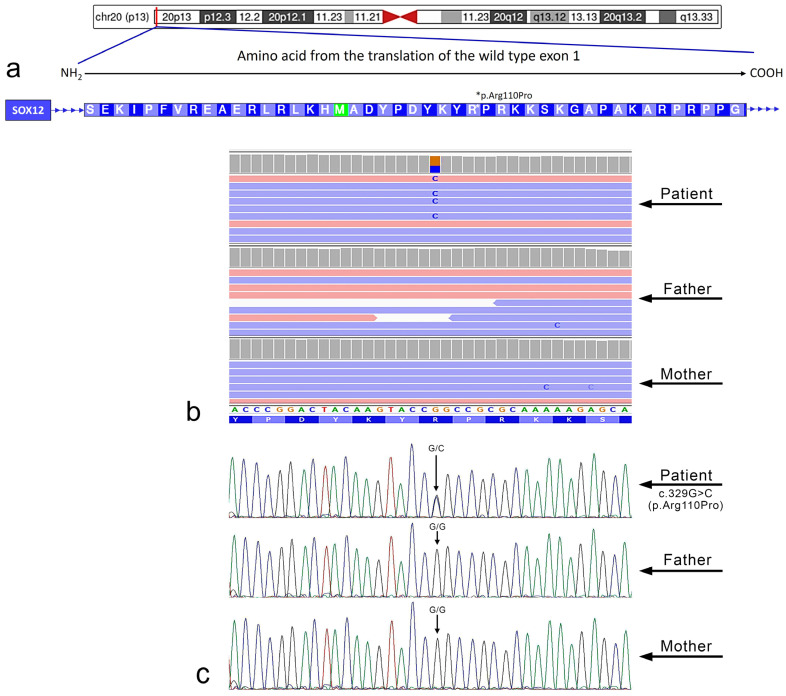
(**a**) Graphical representation of the amino acid sequence corresponding to the region where the mutation was identified within the *SOX12* gene. Additionally, the chromosomal localization of this gene is illustrated. An arrow indicates the NH_2_ → COOH direction of translation for the unique exon of the *SOX12* gene. Figure was a modified from UCSC genome database. The asterisk indicates the variant site. (**b**) Whole exome sequencing results are presented using the Integrative Genomics Viewer (IGV) visualization tool. As shown in the picture, WES was carried out for the examined patient and both the healthy parents. (**c**) Conventional Sanger sequencing was performed to confirm the variant c.329G>C identified by whole exome sequencing (WES). In the electropherograms, the black, blue, green, and red profiles indicate nucleotide G, C, A, and T, respectively.

Whole exome sequencing (WES), performed at the age of 16 years, unveiled the presence of the *de novo* nucleotide variation c.329G>C within the *SOX12* gene (NM_006943). Sanger sequencing confirmed the *de novo* variant. This variation altered the protein sequence leading to the change of the amino acid arginine at position 110 with proline (p.Arg110Pro) (Figure 1). No variants in known genes associated with the patient’s phenotype were detected by the WES analysis.

The in silico predictive analysis involved multiple algorithms, which collectively classified the variant as likely pathogenic (Table 2).

**Figure 2:**
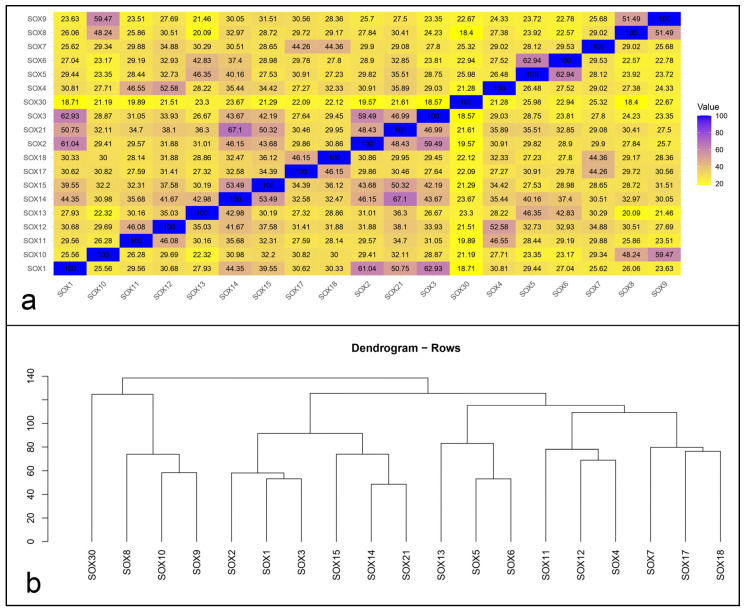
Identity level of 19 SOX genes. (**a**) Heatmap related to the percentage of identity ascertained from the 19 SOX proteins. The percentages were obtained from the Uniprot database. (**b**) Dendrogram obtained considering percentage of identity among *SOX* genes. The percentages of similarity were obtained from the Uniprot database. Both the plots were generated by R studio version 3.6.3.

The DEOGEN2 tool (http://deogen2.mutaframe.com/, accessed on 15 March 2024) indicated a 77.5% residue difference between the wild-type arginine and the mutant proline. Additionally, the mutation was classified as deleterious. The sequence alignment, which compared 19 *SOX* genes (excluding the SRY gene present on the Y chromosome), revealed that *SOX12* shares an identity percentage ranging from 21.51% with *SOX30* to 52.58% with *SOX4*. Based on sequence alignment predictions carried out on the *SOXC* group, *SOX12* shares 46.08% and 52.58% sequence similarity with *SOX11* and *SOX4*, respectively (Figure 2 and Supplementary Figure S1).

**Figure 3:**
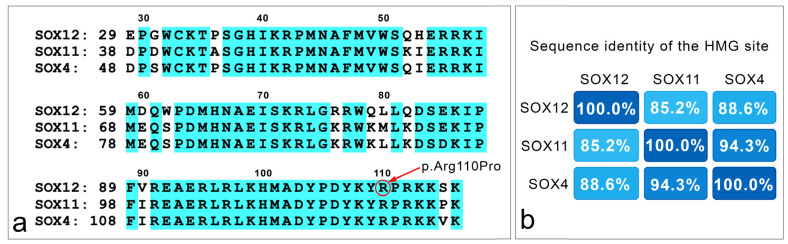
Sequence similarity between HMG sites in *SOXC* genes. (**a**) Sequence alignment of the HMG sites within the three *SOXC* genes, namely *SOX12* (aa 29–116), *SOX11* (aa 38–125) and *SOX4* (48–135). The identity of the residues within the amino acid chain of the three genes is evidenced in light blue. The circled site in *SOX12* sequence indicates the variant amino acid here described (p.Arg110Pro). On the left of each sequence, the number indicates the position in each protein of the first aa here shown. The number in the upper part of the sequences refers to the amino acid sequence of the SOX12 protein. NCBI reference sequences were as follows: NP_008874.2 (*SOX12*), NP_003099.1 (*SOX11*) and 003098.1 (*SOX4*), accessed on 13 May 2024. (**b**) A heatmap depicting the sequence identity of the HMG site within *SOX12*, *SOX11* and *SOX4*. Blue color scale indicates the highest percentage of identity rate (modified from the Uniprot database).

However, when focusing on the high-mobility group (HMG) box region (aa 29 to aa 116 for *SOX12*; aa 38 to aa 125 for *SOX11*; aa 48 to aa 135 for *SOX4*), *SOX12* exhibits high sequence similarity, with 85.2% and 88.6% of similarity, with *SOX11* and *SOX4*, respectively (Figure 3).

**Figure 4:**
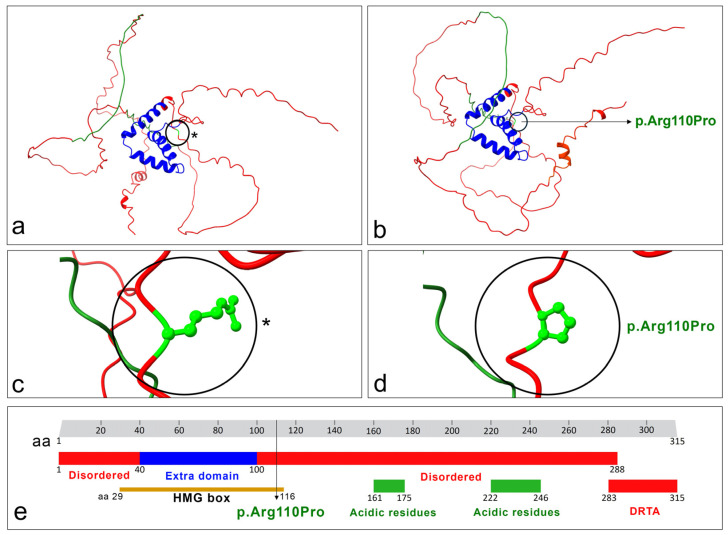
Graphical representation of SOX12 protein with the mutated aa residue. (**a**) Structure prediction of the wild-type SOX12 protein. The black circle indicated by an asterisk shows the wild-type aa at position 110. (**b**) Structure prediction of the mutated SOX12 protein (p.Arg110Pro). The different protein folding between the wild type and the mutated is evident. (**c**) Focus (black circle indicated by an asterisk) on the wild-type amino acid residue (Arg) at position 110. (**d**) Focus (black circle) on the mutated amino acid residue (Pro) at position 110. (**a**–**d**) structures were generated by UCSC ChimeraX v.1.7 software. The variant amino acids (Arg vs. Pro) in the black circles are indicated in green. (**e**) Graphical representation of the SOX12 functional domains, regions and sites. aa: aminocid position. HMG: high-mobility group. DRTA: Domain required for transcriptional activation activity and synergistic coactivation of transcriptional activity with POU3F2. Notably, the mutation occurred within the HMG box (modified from the Uniprot database). The colors vary based on different domains or regions. Specifically, red is used for all regions, blue for the extra domain region and green for both the acidic residues. The mutation site within the protein structure is indicated in light green.

With regards to the impact of the mutation in the protein structure, the analysis carried out by MuPRO indicated that the variant decreased the protein stability, as indicated by the delta delta G score of −0.916 (score ranging from −1 to 1). Conversely, the protein structure prediction analysis, conducted using AlphaFold algorithm, generated five SOX12 protein models. The criteria adopted for the selection of the best model are clearly depicted and described in Figures S2 and S3. Within this context, the analysis of the best model revealed a total of 159 hydrogen bonds in the wild-type SOX12 protein. In contrast, the mutated protein structure prediction identified 103 hydrogen bonds in the “best model” obtained from the analysis. Figure 4 depicts the differences in structure between the wild type (Figure 4a) and the mutated (Figure 4b) SOX12 protein structure. Notably, the changing amino acid residue (p.Arg110Pro) is located at the HMG box site (from aa 29 to aa 116) within the SOX12 protein (Figure 4e).

As depicted in Figure S4, both the predicted Local Distance Difference Test (plDDT) score and the sequence coverage show higher values corresponding to the HMG domain, indicating that it is the most conserved sequence within the SOX12 protein. In fact, the HMG sequence is well covered by many sequences with high sequence identity. This high coverage suggests that the HMG domain is likely conserved, and the mutation identified at position 110 could lead to significant variations in protein folding.

**Figure 5:**
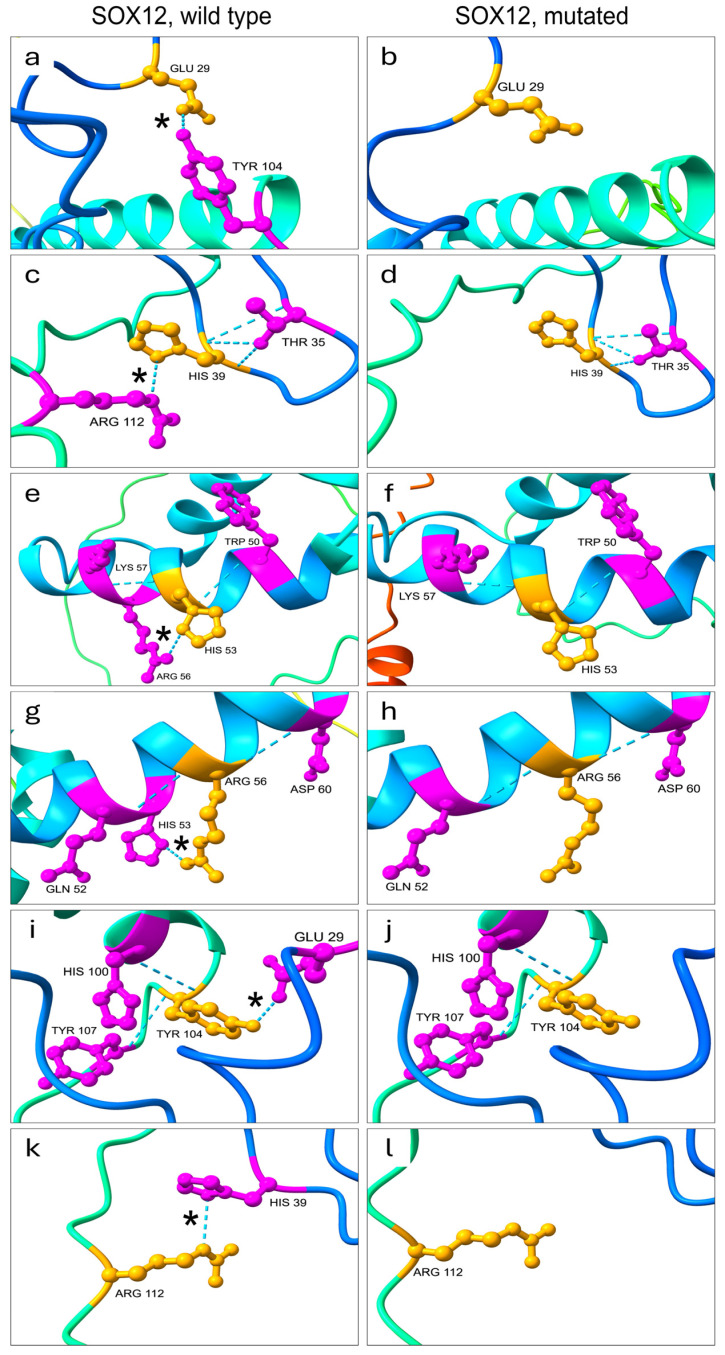
Comparison of the hydrogen bonds between wild-type and mutated SOX12 protein within the HMG domain. Hydrogen bonds are represented by light blue dashed lines. The amino acid (aa) residues considered are colored yellow/orange and the aa residues with which they form hydrogen bonds are colored magenta. In the left panels (**a**,**c**,**e**,**g**,**i**,**k**) hydrogen bonds formed in the SOX12 wild-type protein are shown. In the right panels (**b**,**d**,**f**,**h**,**j**,**l**) hydrogen bonds formed in the SOX12 mutated protein are shown (p.Arg110Pro). The disappearance of the hydrogen bonds between the amino acids Glu29/Tyr104 (panels (**a**/**b**) and (**i**/**j**)), His39/Arg112 (panels (**c**/**d**), and (**k**/**l**)) and His53/Arg56 (panels (**e**/**f**), and (**g**/**h**)) is highlighted by an asterisk in the left images (wild-type SOX12).

Furthermore, the AlphaFold algorithm predicted a variation within the conserved HMG domain affecting hydrogen bonds involving six residues. Specifically, the mutated protein showed a reduction in hydrogen bonds between the residues Glu 29 and Tyr 104, His 39 and Arg 112 and His 53 and Arg 56 (Table 3 and Figure 5).

**Figure 6:**
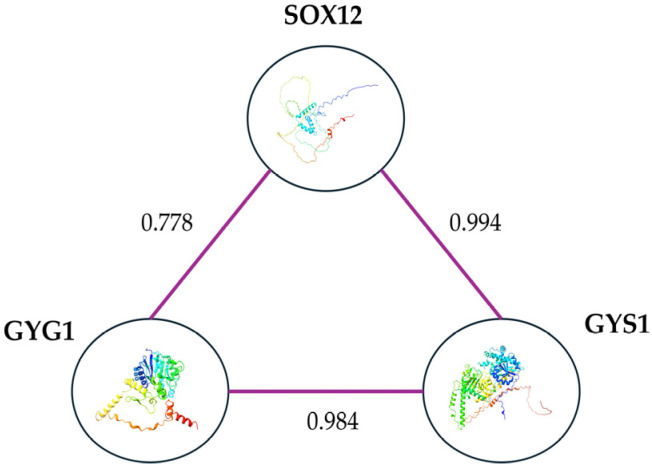
Graphical representation of the protein–protein interaction analysis using the STRING database, supported by experimental evidence. The proteins interacting with *SOX12* include *GYS1* and *GYG1*, with high correlation scores of 0.994 and 0.778, respectively. The correlation score range between 0 and 1 and high values indicate robust correlations.

The analysis involving databases related to protein–protein interaction (STRING) identified a robust interaction between *GYS1* and *GYG1* genes related to glycogen metabolism. As depicted in Figure 6, the interaction between *SOX12*, *GYS1* and *GYG1* is strong and supported by high correlation coefficients.

## 4. Discussion

The current manuscript describes a clinical case of epilepsy in a child born to healthy unrelated parents. The patient experienced frequent epileptic seizures, alongside neurodevelopmental delay and childhood emotional behavior disorder. WES trio analysis revealed a novel *de novo* variant within the *SOX12* gene. Notably, no mutations were detected in genes previously earmarked as candidates for epilepsy. The variant was not reported in ExAC nor 1000 G databases. According to the ACMG criteria, the variant was described as likely pathogenic. The protein structure prediction analysis carried out by UCSF ChimeraX revealed a structure modification from the wild-type *SOX12* to the mutated form. Specifically, the structure prediction revealed the loss of 56 hydrogen bonds from the wild type to the mutated form. Additionally, MuPRO indicated that the variant significantly decreased protein stability. Furthermore, DEOGEN2 indicated a 77.5% residue difference between the wild-type arginine and the mutated proline residue. It is worth noting that various algorithms, specifically EIGEN, Mutation Assessor, MutPred2 and MVP, suggest that the variant is benign. However, we emphasize that the potential pathogenic role of this variant is also based on other factors, such as the strong association of *SoxC* genes with developmental issues, despite the “uncertainty” indicated by the in silico predictors.

Based on these predictions, we hypothesize that the amino acid change significantly impacted protein function, hypothesizing an autosomal dominant (AD) inheritance pattern. Indeed, all the OMIM-annotated *SOX* genes display an AD inheritance model.

As has been extensively documented, *SOX* gene family is intricately involved in the neurodevelopmental process. Specifically, disruptions of *SOX1*, *SOX2*, *SOX4* and *SOX11* have been found to be intricately involved in the onset of epileptic seizures [18,19,20,21,22,55]. Furthermore, *SOX4* and *SOX11*, members of the *SoxC* gene group, are both associated with neurodevelopmental delay and intellectual disability [55,56,57]. Additionally, about 58% of the 19 *SOX* genes have been assigned a MIM phenotype number. Interestingly, within this group, 73% of the genes with a MIM phenotype number are associated with clinical features related to neurodevelopmental disorders.

The *SOX12* gene is part of the *SOX* gene family. In this context, protein sequence similarity analysis reveals that *SOX12* shares a percentage of its sequence identity with all members of the *SOX* gene family, ranging from 21.51% with *SOX30* to 52.58% with *SOX4*. Remarkably, the mutation identified within the *SOX12* gene is situated within the high-mobility group (HMG) site. Within this context, the HMG domain of *SOX12* shares 85.2% and 88.6% of its sequence identity with the same domains in *SOX11* and *SOX4*, respectively, both belonging to the *SoxC* group. As extensively documented, the functional HMG site is crucial for binding DNA molecules at their specific binding sites [58]. This functional domain is associated with specific gene ontology (GO) terms (GO:0003677), corresponding to “DNA binding”. The high percentage of identity within the HMG domains underscores its remarkable conservation rate, suggesting that the variant that we observed (p.Arg110Pro) may disrupt its function as a DNA binding site. In fact, both PhastCons100way and PhyloP100way scores indicate the high conservation rate (1 and 4.124 respectively) of the specific mutation site, being conserved among 100 vertebrate species. Furthermore, the AlphaFold algorithm predicts that both the plDDT score and sequence coverage are highest in the HMG domain, indicating it is highly conserved within the SOX12 protein. This suggests that a mutation at position 110 could significantly impact protein folding (Figure S4).

As documented, *SOX12* demonstrates overlapping embryonic expression patterns and DNA binding characteristic with *SOX4* and *SOX11*, all of which belong to the C group of the *Sox* gene family (SoxC). They are all expressed in post-mitotic neurons and play crucial roles in neuronal maturation during embryonic neurogenesis. *SOX12* is essential for organogenesis, guiding the transition of embryonic stem cells into multipotent cells, but is not detected in the adult mouse brain [25,31,32,33,34,36]. Although no neurodevelopmental disorders have been directly attributed to defects in the *SOX12* gene thus far, it has been implicated as a candidate gene in developmental delay. Specifically, *SOX12* was identified in a microdeletion of 20p13 alongside another gene, highlighting its potential role in neurodevelopmental processes [35].

According to previous studies and the expression databases, *SOX12* is predominantly expressed in human nervous tissues [59]. Specifically, as indicated, this gene shows the highest expression pattern during fetal development [59]. Specifically, the highest expression patterns of *SOX12* gene have been observed in the cerebellum, followed by the cerebral cortex tissues. Within this context, it is worth underscoring that dysfunctions within these tissues are significantly associated with epilepsy [60,61,62,63].

*SOX12* is a transcription factor with nuclear localization [59]. Within this context, it is worth mentioning that several transcription factors involved in DNA binding are intricately associated with both neurodevelopmental and psychiatric disorders [64,65,66].

Furthermore, as reported by the STRING, Biogrid and IntAct databases, alongside findings from an interactome study [67], the pathway analysis uncovered a protein–protein interaction with the *GYS1* and *GYG1* genes. In particular, the *Glycogen Synthase 1* (*GYS1*) gene stimulates glycogen biosynthesis, and its dysregulation has been associated with epilepsy in mouse models, impacting synaptic plasticity [68,69,70]. It has a MIM phenotype number associated with defects in glycogen storage (611556) but has also been recently described in the literature as a potential candidate for Lafora disease, a progressive myoclonic epilepsy [71,72,73]. As is well known, brain glycogen acts as an energy reserve during hypoxia, aiding neuronal function. Dysregulated metabolism may heighten epilepsy susceptibility by disrupting energy balance and neuronal excitability [68,69,74,75]. Additionally, the *GYG1* gene is associated with defects in glycogen storage (613507) and polyglucosan body myopathy (616199).

This study addresses a critical gap in the existing literature by investigating the role of *SOX12* in neurodevelopmental disorders. Through our research, we aim to provide essential insights into the genetic underpinnings of epilepsy and related conditions, ultimately advancing our understanding and paving the way for future therapeutic strategies. We emphasize that functional analyses are needed to confirm the involvement of *SOX12* gene in neurogenesis. Additionally, we highlight that mutations in this gene may contribute to a spectrum of neurodevelopmental disorders, including epilepsy and borderline personality disorder, as observed in the patient examined in this study. The objective of this research is to provide groundwork for assigning a MIM phenotype number to this gene, aligning with the precedent set by other *SoxC* genes such as *SOX4* and *SOX11*.

## 5. Conclusions

The current manuscript presents a clinical case of a patient presenting with epilepsy, as well both neurodevelopmental and borderline personality disorders. WES trio analysis revealed a *de novo* variant within *SOX12*, a transcription factor crucial for DNA binding within the C group of the SOX family. As previously documented, this gene is predominantly expressed in brain tissues, and notably, except for *SOX12*, all the members of the C group have an entry code assigned to neurodevelopmental disorders. The variant was situated within the critical high-mobility group (HMG), a functional protein region crucial for DNA binding. In silico predictions described the variant as likely pathogenically significant, underscoring significant structural variations affecting protein function. Further analyses are imperative to confirm the involvement of this gene in neurogenesis and to assess the impact of the identified mutation in this study.

## Figures and Tables

**Table 1:** Criteria used for the classification of the observed nucleotide variation ^(a)^.

Evidence of Pathogenicity	Category Code	Description
Strong	PS2	*De novo* (both maternity and paternity confirmed) in a patient with the disease and no family history.
Moderate	PM2	Absent from controls (or at extremely low frequency if recessive) in Exome Sequencing Project, 1000 Genomes Project, or Exome Aggregation Consortium.
Moderate	PP4	Patient’s phenotype or family history is highly specific for a disease with a single genetic etiology.
Germline Variant Classification (ACMG criteria): Likely Pathogenic		

^(a)^ According to the American College of Medical Genetics (ACMG) guidelines [46].

**Table 2:** Multiple in silico prediction algorithms for predicting the effect of the variant c.329G>C within the *SOX12* gene (NM_006943).

Tool	Prediction	Score
CADD	Uncertain	23.8999
M-CAP	Pathogenic Moderate	0.979
PrimateAI	Pathogenic, moderate	0.9664
EIGEN	Benign, moderate	−0.2651
Mutation assessor	Benign, moderate	0.55
MutationTaster	Disease-causing	0.9626
PROVEAN	Pathogenic-supporting	−5.77
SIFT	Pathogenic-supporting	0
FATHMM-MKL	Pathogenic-supporting	0.84340
MutPred2	Benign-supporting	0.464
MVP	Benign-supporting	0.6871
DANN	Uncertain	0.9784
FATHMM	Damaging	−3.33
LRT	Uncertain	0.000318
SIFT4G	Uncertain	0.004
DEOGEN2	Deleterious	0.6900
BayesDel addAF	Uncertain	0.1551
BayesDel noAF	Uncertain	−0.0149
MetaLR	Uncertain	0.5195
MetaRNN	Uncertain	0.5739
MetaSVM	Uncertain	0.02229

**Table 3:** Variation in the hydrogen bond numbers between the wild-type and mutated SOX12 protein, within the HMG domain.

aa Residue	HyB aa Partner ^(a)^	SOX12^wt^ (Arg 110) ^(b)^	SOX12^mut^ (Pro 110) ^(c)^
Glu 29	Tyr 104	1	0
His 39	Thr 35	3	3
	Arg 112	1	0
His 53	Trp 50	1	1
	Arg 56	1	0
	Lys 57	1	1
Arg 56	Gln 52	1	1
	His 53	1	0
	Asp 60	1	1
Tyr 104	Glu 29	1	0
	His 100	1	1
	Tyr 107	1	1
Arg 112	His 39	1	0

^(a)^ Amino acid (aa) involved in hydrogen bonds (HyB) with the aa residue listed in the first column; ^(b)^ number of hydrogen bonds in the wild-type SOX12 protein; ^(c)^ number of hydrogen bonds in the mutated SOX12 protein.

## Data Availability

The data presented in this study are available on request from the corresponding author.

## Supplementary Materials

The following supporting information can be downloaded at: https://www.mdpi.com/article/10.3390/cimb46070383/s1, Figure S1: Sequence alignment of the *SOX* genes; Figure S2: Models of the protein structure prediction; Figure S3: Heatmaps of the AlphaFold prediction; Figure S4: Prediction of the most conserved region.
