# Supplementary material for: Implications of a De Novo Variant in the SOX12 Gene in a Patient with Generalized Epilepsy, Intellectual Disability, and Childhood Emotional Behavioral Disorders
Source: Curr Issues Mol Biol. 2024 Jun 26;46(7):6407–22. doi: 10.3390/cimb46070383 (PMC11276073; doi:10.3390/cimb46070383)
Supplement: Supplementary file 1 [file cimb-46-00383-s001.zip › cimb-3034749-supplementary.pdf]

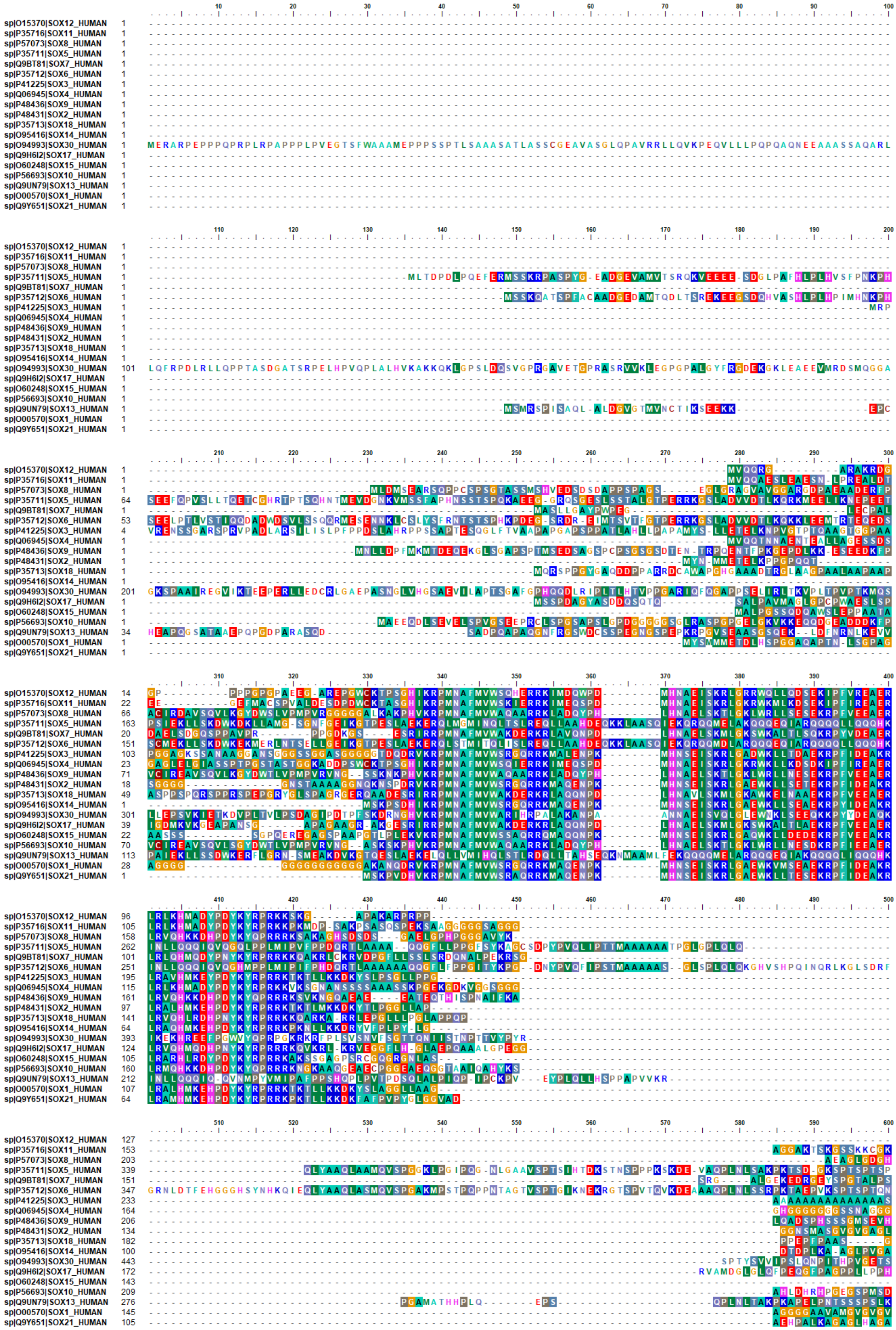

sp|O15370|SOX12_HUMAN
sp|P35716|SOX11_HUMAN
sp|P57073|SOX8_HUMAN
sp|P35711|SOX5_HUMAN
sp|Q9BT81|SOX7_HUMAN
sp|P35712|SOX6_HUMAN
sp|P41225|SOX3_HUMAN
sp|Q06945|SOX4_HUMAN
sp|P48436|SOX9_HUMAN
sp|P48431|SOX2_HUMAN
sp|P35713|SOX18_HUMAN
sp|O95416|SOX14_HUMAN
sp|O94993|SOX30_HUMAN
sp|Q9H6I2|SOX17_HUMAN
sp|O60248|SOX15_HUMAN
sp|P56693|SOX10_HUMAN
sp|Q9UN79|SOX13_HUMAN
sp|O00570|SOX1_HUMAN
sp|Q9Y651|SOX21_HUMAN

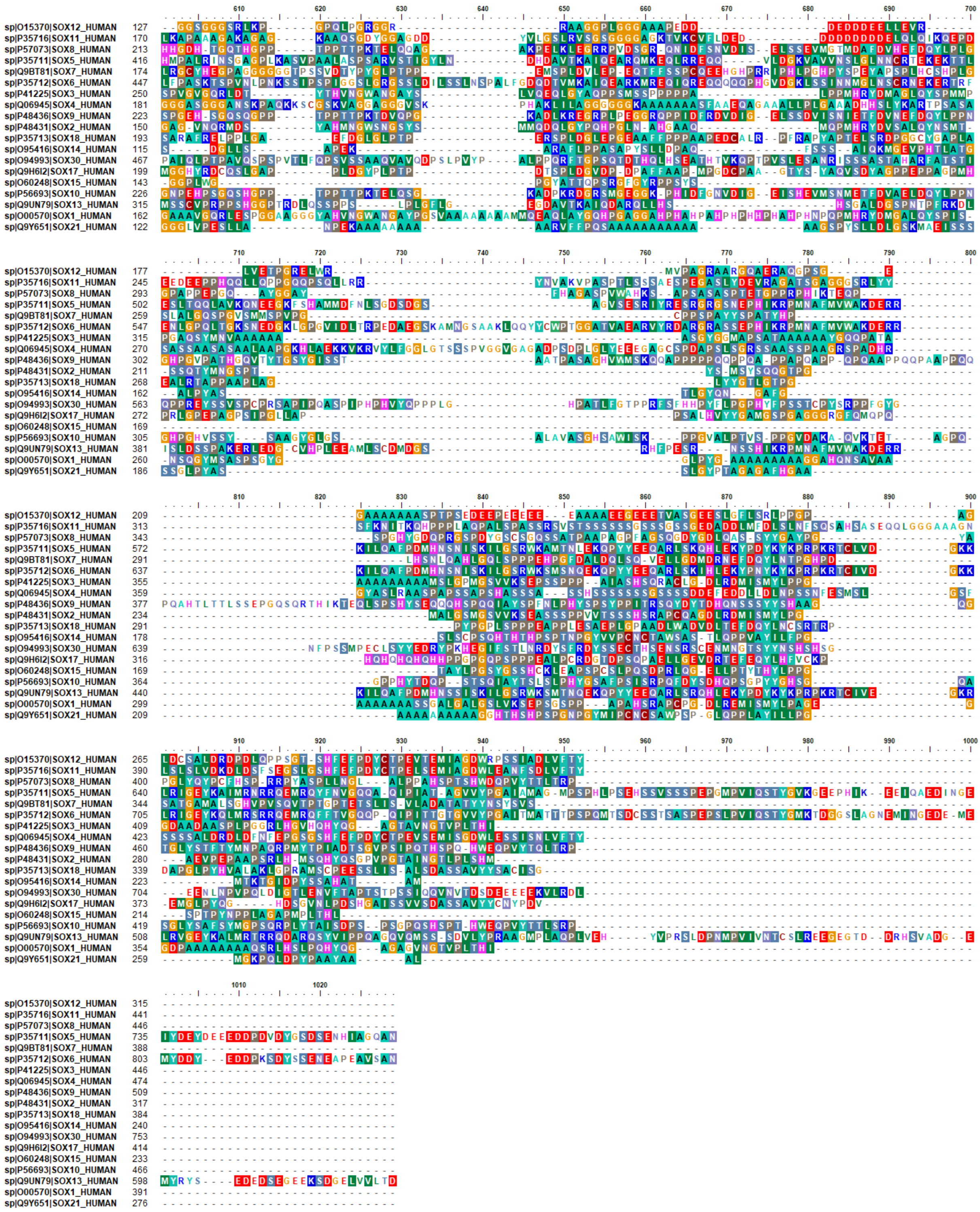

Figure S1. Sequence alignment related to the 19 SOX genes (All the SOX genes, excluding SRY gene, present in the Y chromosome). The analysis was conducted using BioEdit software version 7.2 using the whole protein sequences.

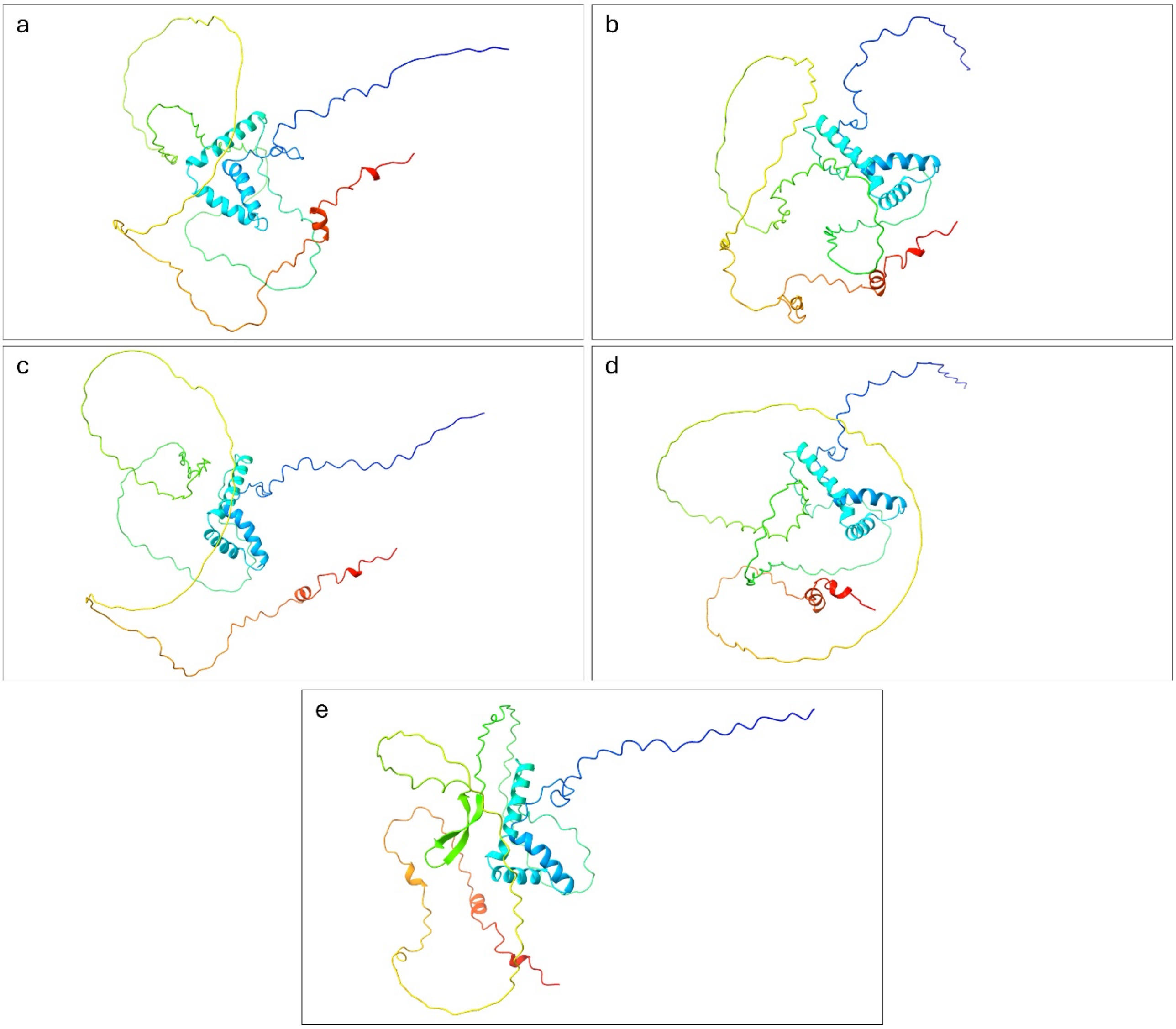

Figure S2. Five models generated as results of the protein structure prediction using AlphaFold algorithm, employing the software UCSF ChimeraX. Rainbow coloring has been applied to highlight the progression of amino acid residues from the N-terminus to the C-terminus of the protein chain. Typically, the N-terminus is depicted in blue and progresses through the rainbow colors to red at the C-terminus. This aids in visually tracing the sequence of the protein and comprehending its folding and structure. The model that we adopted for the study was labelled in the Figure as "a", consistently with the AlphaFold algorithm that identify "a" as the "best model".

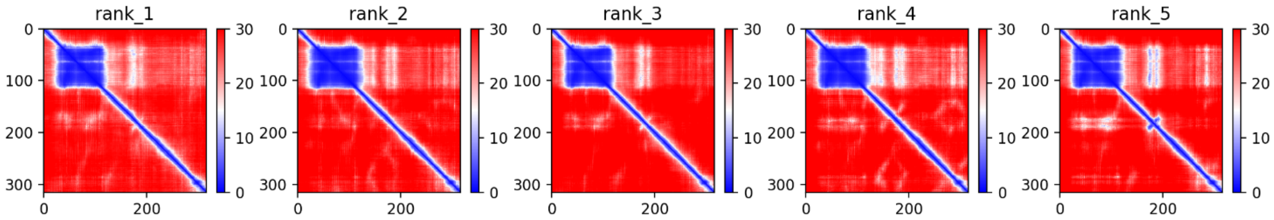

Figure S3. Heatmaps generated as results of the AlphaFold prediction for determining the best predicted model. Specifically, the diagonal line from the bottom-left to the top-right represents the proximity of residues to themselves, which is always close and thus should be present in all maps. Blue regions indicate contacts between residues. Larger and more defined blue regions typically indicate a higher confidence in the structural prediction for those parts of the protein. Red regions indicate areas where there is no contact between residues. Within this context, rank_1 shows a prominent blue region between residues 0-100, indicating strong contacts. It has a clean and defined pattern with few artifacts (white noise), suggesting a reliable structure. Furthermore, rank_2 is quite similar to rank_1 but with some additional blue regions around the same area, which might be slightly less clean than rank_1. Rank_3 shows more white noise and less defined blue regions compared to rank_1 and rank_2, suggesting lower confidence in these areas. Rank_4 has defined blue regions but a wide array of white noise, making it less clean. rank_5 shows an additional distinct blue line and some noise, suggesting possible alternative contact points, but overall, less clean than rank_1. Overall, consistently to the AlphaFold prediction, rank_1 appears to be the best model because it has the most defined and clean blue regions, indicating strong and confident predictions of residue contacts. This implies that the structure predicted by the rank_1 model is likely to be the most accurate and reliable among the five shown.

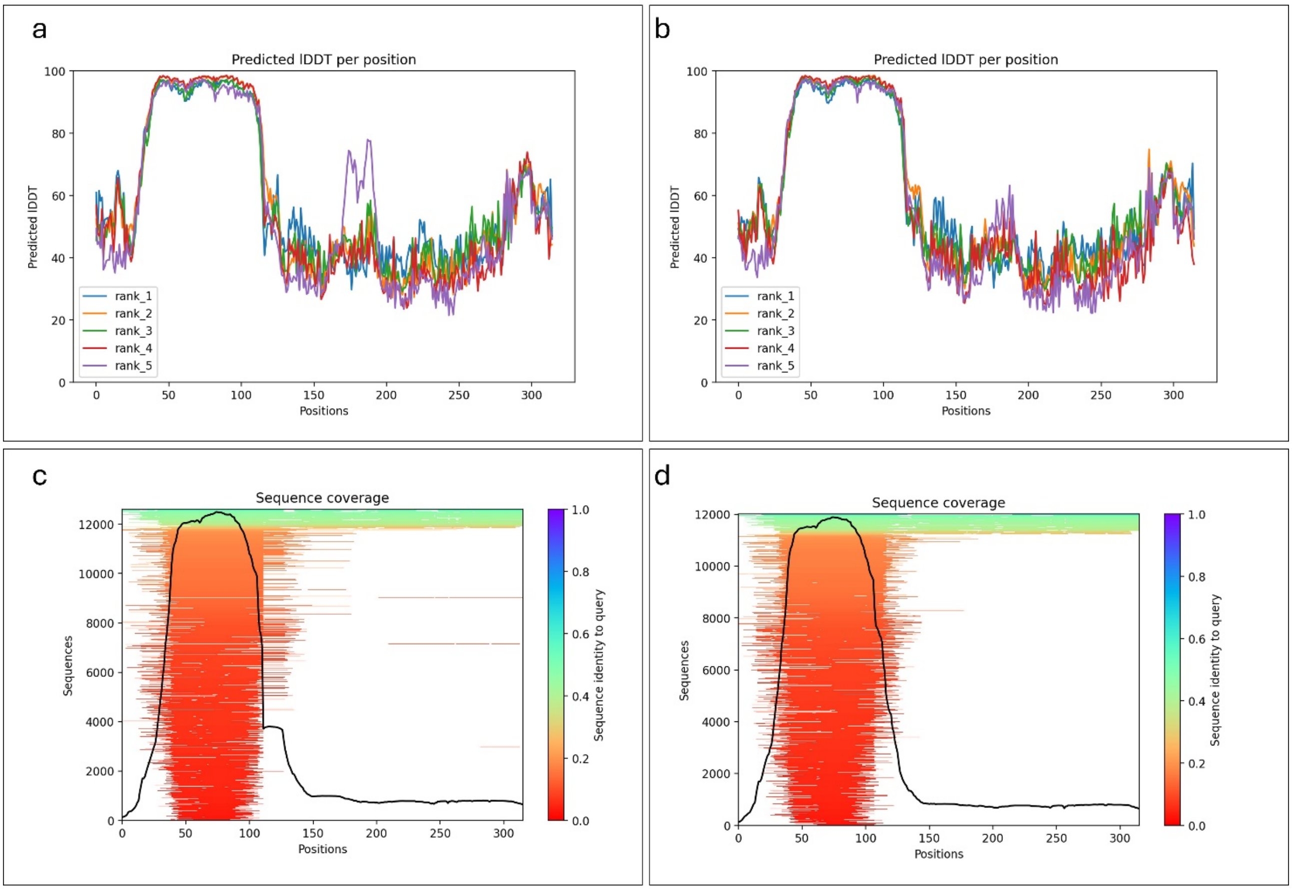

Figure S4. Prediction of the most conserved region by the plDDT scores and the sequence coverage. Figures a and b illustrate the variation in the predicted Local Distance Difference Test (plDDT) scores, a confidence metric used in protein structure prediction, particularly with AlphaFold, employing UCSF ChimeraX. The plDDT score, ranging from 0 to 100, measures the confidence or accuracy of the predicted protein structure at a local, residue-by-residue level, with higher scores indicating greater confidence. As shown in figures a and b, the high mobility group (HMG) domain (from aa 16 to aa 116) stands out as the most conserved region. Notably, in the wild-type plot (a), there is a moderately conserved spot between residues 150 and 200, which is absent in the mutated prediction (b). Figures c and d display sequence coverage plots, commonly used in sequence alignment or homology modeling. The x-axis represents the position of the amino acids in the query sequence (with c representing the wild type and d representing the mutated model). Each number corresponds to a specific residue position or range of positions in the query sequence. The y-axis represents the number of sequences aligned to the query sequence. The black line likely represents the average or cumulative coverage across these positions, indicating the density or number of sequences aligning at each position. In both c and d, the central red area spanning the HMG domain has the highest density of aligned sequences (indicated by the black line), suggesting that these positions are well-covered by many sequences with high sequence identity. This high coverage suggests that the HMG domain is likely conserved, and the mutation identified at position 110 could lead to significant variations in protein folding.
